# Access site complications after transfemoral aortic valve implantation - a comparison of Manta and ProGlide

**DOI:** 10.1186/s42155-018-0026-0

**Published:** 2018-09-21

**Authors:** Pavel Hoffmann, Ahmed Al-Ani, Thomas von Lueder, Jenny Hoffmann, Peter Majak, Ove Hagen, Helga Loose, Nils Einar Kløw, Anders Opdahl

**Affiliations:** 1 Department of Cardiology, Section for Interventional Cardiology, Division of Cardiovascular and Pulmonary Diseases, Oslo University Hospital, Ullevål, Oslo, Norway; 2 Department of Cardiology B, Division of Medicine, Oslo University Hospital, Ullevål, Oslo, Norway; 3 The Lundberg Laboratory, Sahlgrenska Academy, University of Gothenburg, Gothenburg, Sweden; 4 Department of Cardiothoracic Surgery, Division of Cardiovascular and Pulmonary Diseases, Oslo University Hospital, Ullevål, Oslo, Norway; 5 Department of Anesthesiology, Division of Emergencies and Critical care, Oslo University Hospital, Ullevål, Oslo, Norway; 6 Department of Vascular Diseases, Section for Vascular Surgery, Division of Cardiovascular and Pulmonary Diseases, Oslo University Hospital, Oslo, Norway; 7 Department of Radiology, Section for Interventional Radiology, Division of Radiology and Nuclear Medicine, Oslo University Hospital, Ullevål, and Institute for Clinical Medicine, University of Oslo, Oslo, Norway

**Keywords:** Transfemoral TAVI, Vascular closure device, ProGlide, Manta, Access site complications

## Abstract

**Background:**

Despite decreasing sheath diameter, access site bleeding and vascular complications are still a major concern in transfemoral aortic valve implantation (TAVI), and may increase morbidity and even increase mortality. The aim was to compare safety of arterial closure in transfemoral TAVI with two different principles, pre-suture with ProGlide and collagen plug closure with Manta.

**Results:**

Seventy-six patients treated with ProGlide and 75 with Manta were analysed. The endpoints were 1: access site vascular complications and 2: non-planned vascular or endovascular surgery at the puncture site. Complications occurred in 2 (2.7%) ProGlide and in 8 (10.7%) Manta cases, *p* = 0.047. During the learning phase there were no significant differences. In the established phase there was one event (2%) in the ProGlide group, compared to 6 endpoints (12.0%), *p* = 0.047, in the Manta group.

Unplanned surgery or intervention was seen in two (2.7%) ProGlide and in 7 (9.3%) Manta patients, p = ns. There were no significant differences during the learning phase. In established use, endpoints occurred more frequently in patients treated with the Manta device (12%), than in patients treated with the ProGlide (2%), *p* = 0.047.

**Conclusion:**

The ProGlide presuture closure device was associated with significantly lower rates of vascular complications and lower rates of surgery and interventions compared to the collagen plug Manta system.

**Trial registration:**

The data were collected from Internal quality control registry on treatment of patients with valvular heart disease with or without coronary artery disease, No 2014/17280, Oslo University Hospital, Ullevål.

## Background

In recent years there has been a development towards a minimalistic approach to transfemoral transcatheter aortic valve implantation (TF-TAVI). In truly simplified TF-TAVI, percutaneous vascular closure devices (VCD) are used instead of surgical vascular access and closure, even when large diameter arterial access of 14–22 French (Fr) has been used (Babaliaros et al. 2014; McCabe et al. 2016). Despite decreasing sheath diameter, access site bleeding and vascular complications in TAVI are still a major concern and may increase morbidity, like acute kidney injury, and even increase mortality (Nara et al. 2017; Dencker et al. 2016; Mangla and Gupta 2016). There is a great impact of puncture site complications on the procedure outcome in the frequently fragile TAVI patients, and thus, successful percutaneous closure of the large bore access site is of major significance.

The aim of the present study was to investigate the efficacy of two mechanically distinct VCD principles, a collagen plug based post procedure closure device (Manta), to a percutaneous pre-suture mediated closure device (ProGlide). ProGlide is a well-established and well-documented VCD used in many centres worldwide (Barbash et al. 2015; Maniotis et al. 2017), while the Manta device recently was introduced to Europe (Van Gils et al. 2016; Van Mieghem et al. 2017).

## Methods

### Patients

The present study includes 157 consecutive patients scheduled for TAVI in a newly established TAVI centre at Oslo University Hospital, Ullevål, Norway, with intention of performing percutaneous mini invasive TF-TAVI. Six patients were excluded from the present study due to the following causes; subclavian artery access (*n* = 3), surgical femoro-iliac cut-down (*n* = 2), and one patient died during the procedure. The remaining 151 patients were treated between December 2015 and March 2018. All patients were classified by the Society of Thoracic Surgeons/American College of Cardiology transcatheter aortic valve replacement (STS/ACC TAVR) risk score (Arsalan et al. 2018).

All femoral punctures intended for large bore access were preplanned from CT angiography and access was obtained under fluoroscopy using a J-tip wire in the common femoral artery (CFA) at the puncture site, either introduced by a contralateral or an ipsilateral peripheral puncture. Ultrasound guidance was not systematically used. All punctures were made by the same three experienced interventionists (AA-A, AO or PH).

When applicable, anticoagulants were stopped three days prior to TAVI procedure. All patients received intravenous Heparin and activated clotting time (ACT) was targeted to 250–300 s. Vascular closing with both ProGlide and Manta were started after reversing the Heparin effect with intravenous Protamin 50 mg. For both VCDs the initial procedures were performed with a proctor or device specialist, as recommended by the respective vendor.

### Vascular closure devices

The Perclose ProGlide (Abbot Vascular, USA) is a 6 Fr suture mediated closure system developed for closure of femoral artery punctures up to 8 Fr. When using larger access site diameters, 14–24 Fr, 2 ProGlide can be used in the same puncture site. The 2 ProGlides are placed as presutures with an angle of 30–45 degrees to each side. After completion of the TF-TAVI procedure the large bore sheath is removed and the preformed sutures are advanced and tightened to obtain hemostasis (Maniotis et al. 2017).

The Manta (Essential Medical Inc., USA) is a collagen based vascular plug for post procedure closure of large bore access sites. Briefly, the Manta VCD consists of a resorbable intraarterial toggle and an extravascular hemostatic collagen plug and a suture and a stainless steel suture lock which keeps the toggle and the collagen plug together, on each side of the arterial wall. The Manta components resorb within 6 months, but if earlier reintervention would be needed the puncture is clearly indicated by the stainless steel suture lock and can thus be avoided. The Manta is delivered in two sizes, 14 and 18 Fr, for punctures of 10–22 Fr (Van Gils et al. 2016).

### Study endpoints

This study focuses on ipsilateral large bore arterial access site complications during index hospitalization, which with a reasonable probability can be attributed to failure of the vascular closure device used. Bleedings included corresponded to Valve Academic Research Consortium (VARC)-2 “Major bleeding” while other complications like pseudoaneurysm best correspond to VARC-2 “Minor vascular complications” (Kappetein et al. 2012).

The endpoints were 1: vascular complications including bleeding, occlusion, flow limiting high grade stenosis or dissection, pseudoaneurysm or other complications directly attributable to the puncture site or the VCD used or 2: non-planned vascular surgery and / or use of endovascular stent or stentgraft or other endovascular intervention at the puncture site.

The separate components of the endpoints as bleeding, pseudoaneurysm, occlusion, flow limiting high grade stenosis or dissection at the puncture site and separate interventions as vascular surgery and / or use of endovascular stent or stentgraft or other endovascular intervention at the puncture site are also presented.

### Study design

All eligible patients from one TAVI center were included in a non-randomized registry and analysed retrospectively. All patients were treated using self-expanding bioprostheses from Medtronic (Medtronic, USA); CoreValve, Evolut R or Evolut PRO. Initially, all 14–20 Fr closures were performed using the ProGlide. Sixty-nine consecutive patients were treated using the ProGlide VCD. In May 2017 the first TAVI procedure using Manta VCD was performed. Subsequently, all patients but seven have consecutively been treated using Manta. Of the seven later, non-consecutive ProGlide procedures four were performed due to availability and three due to operator preferences.

The ProGlide and the Manta groups have been compared for endpoints in the total cohort of patients. Further, both groups were divided into the first 25 patients with the respective VCD to access the learning period complications and into the remaining patients (51 for ProGlide and 50 for Manta) to assess the results during established use of the device.

### Vessel characteristics

The diameter and calcification was evaluated from contrast enhanced multislice computed tomography (CT) images performed as pre-procedural planning for the TAVI procedure. Smallest lumen diameter was measured in two planes in the external iliac artery and in the common femoral artery (CFA). The mean of these diameters is used as minimal diameter in the respective segment. Calcification was visually graded as none or mild (=0) or moderate or severe (=1). Measurements of lumen diameter and calcium scoring were performed by an external observer not involved in the choice of VCD. Procedure planning and choice of introducers was based on CT angiography. Procedures were performed using the Medtronic Sentrant sheath. The mean of the CFA inner diameter was also related to the sheath size to calculate a CFA/sheath size ratio.

### Statistical considerations

All data are obtained retrospectively from a consecutive, non-randomized registry at a single TAVI center. In the endpoints, only the first complication and only the first treatment were included. If present, other vascular access site complications and treatments are presented in results but are not statistically analysed. Data were analysed using Excel (Microsoft, USA). Continuous data are presented as mean ± standard deviation (SD) and categorical variables are presented as number and percentages. Student’s t-test (two sided, non-paired observations) was used for analysis of continuous variables. Categorical variables were analysed by the Kji-square test. For both, *p*-value of < 0,05 was considered as significant.

## Results

A total of 151 continuous patients accepted for TF-TAVI were included. In 76 patients ProGlide was used for closure of large bore vascular access and in 75 patients Manta was used. Mean age was 81 ± 8 years and the STS/ACC TAVR score was 2.8 ± 1.0. Mean echocardiographic transaortic valve gradient was 53 ± 16 mmHg and aortic valve area was 0.6 ± 0.2 cm^2^. Baseline characteristics of the respective groups are presented in Table 1. There were no significant differences between groups, except for a greater proportion of women and higher degree of pelvic artery calcification in ProGlide compared to the Manta group.

**Table 1:** Baseline characteristics

	ProGlide	Manta	p
Number of patients	76	75	
Age (years) mean ± SD	80.8 ± 8.3	81.2 ± 6.5	0.728
Female, n (%)	46 (60.5)	33 (44.0)	0.042
BMI (kg/m^2^)	25.6 ± 4.7	25.7 ± 4.3	0.900
Diabetes, n (%)	14 (18.4)	15 (20.0)	0.805
Hypertension, n (%)	53 (69.7)	54 (72.0)	0.760
Prior PCI, n (%)	23 (30.3)	26 (34.7)	0.818
Prior CABG, n (%)	10 (13.2)	7 (9.3)	0.457
Kreatinin (μmol/L)	97.0 ± 33.9	97.3 ± 27.6	0.947
eGFR (CKD-EPI, mL/min/1.73 m2)	58.2 ± 19.7	59.8 ± 17.4	0.604
STS/ACC TAVR score (mean ± SD)	2.9 ± 1.0	2.8 ± 1.0	0.546
*Echocardiographic parameters:*			
LVEF (%) mean ± SD	54.2 ± 6.5	52.0 ± 11.2	0.138
Mean aortic valve gradient (mmHg) mean ± SD	53.7 ± 15.6	51.4 ± 15.5	0.370
Aortic valve area (cm^2^) mean ± SD	0.7 ± 0.2	0.6 ± 0.2	0.403
*Anatomic data, minimal luminal diameter:*			
Common femoral artery (mm) mean ± SD	7.8 ± 1.4	8.1 ± 1.5	0.247
External iliac artery (mm) mean ± SD	8.2 ± 1.3	8.1 ± 1.4	0.815
Calcification ≥ moderate, n (%)	48 (63.2)	32 (42.7)	0.012

*SD* Standard Deviation, *BMI* Body Mass Index, *PCI* Percutaneous Coronary Intervention, *CABG* Coronary Artery Bypass Graft, *eGFR* estimated Glomerular Filtration Rate, *STS/ACC* Society of Thoracic Surgeons/American College of Cardiology, *TAVR* Transcatheter Aortic Valve Replacement *LVEF* Left Ventricular Ejection Fraction

Table 2 details baseline characteristics for the learning phase (patient 1–25) and for the established use phase (patient > 25), in the respective vascular closure device. The only significant differences were the larger CFA-diameter (*p* = 0.019) and presence of less pelvic artery calcification (*p* = 0.022) in the established Manta-use group as compared to the established ProGlide-group. However, the CFA / sheath size ratio was not significantly different between these groups, *p* = 0.074.

**Table 2:** Baseline characteristics learning and established method in ProGlide and in Manta VCD

	ProGlide learning	Manta learning	p	ProGlide established	Manta established	p
Number of patients	25	25		51	50	
Age (years) mean ± SD	81.2 ± 7.6	80.9 ± 7.1	0.893	80.6 ± 8.7	81.3 ± 6.3	0.612
Female, n (%)	18 (72.0)	12 (48.0)	0.083	28 (54.9)	21 (42.0)	0.195
BMI (kg/m^2^)	24.1 ± 5.1	24.7 ± 4.7	0.710	26.3 ± 4.4	26.2 ± 4.0	0.897
Diabetes, n (%)	2 (8.0)	4 (16.0)	0.384	12 (23.5)	11 (22.0)	0.854
Hypertension, n (%)	15 (60.0)	18 (72.0)	0.370	38 (74.5)	36 (72.0)	0.776
Prior PCI, n (%)	8 (32.0)	5 (20.0)	0.333	15 (29.4)	21 (42.0)	0.186
Prior CABG, n (%)	1 (4.0)	2 (8.0)	0.551	9 (17.6)	5 (10.0)	0.266
Kreatinin (μmol/L)	98.0 ± 27.7	101.8 ± 32.4	0.657	96.5 ± 36.9	95.1 ± 24.9	0.823
eGFR	57.0 ± 19.9	60.0 ± 20.1	0.645	60.0 ± 20.1	61.1 ± 16.7	0.753
(CKD-EPI, mL/min/1.73 m2)						
STS/ACC TAVR score	3.0 ± 1.0	3.0 ± 1.3	0.904	2.8 ± 1.0	2.6 ± 0.8	0.359
(mean ± SD)						
*Echocardiographic parameters:*						
LVEF (%) mean ± SD	55.1 ± 6.0	50.9 ± 12.8	0.151	53.8 ± 6.8	52.5 ± 10.4	0.464
Mean aortic valve gradient	54.0 ± 11.2	49.3 ± 16.5	0.265	53.5 ± 17.4	52.3 ± 15.1	0.717
(mmHg) mean ± SD						
Aortic valve area (cm^2^)	0.6 ± 0.2	0.6 ± 0.1	0.964	0.7 ± 0.2	0.6 ± 0.2	0.331
mean ± SD						
*Anatomic data, minimal luminal diameter:*						
Common femoral artery (mm)	8.1 ± 1.2	7.5 ± 1.5	0.1462	7.7 ± 1.5	8.4 ± 1.4	0.019
mean ± SD						
External iliac artery (mm)	8.3 ± 1.4	7.7 ± 1.4	0.140	8.1 ± 1.2	8.3 ± 1.4	0.420
mean ± SD						
Calcification ≥ moderate, n (%)	17 (68.0)	13 (52.0)	0.248	31 (60.8)	19 (38.0)	0.022

*SD* Standard Deviation, *BMI* Body Mass Index, *PCI* Percutaneous Coronary Intervention, *CABG* Coronary Artery Bypass Graft, *eGFR*; estimated Glomerular Filtration Rate, *STS/ACC* Society of Thoracic Surgeons/American College of Cardiology, *TAVR* Transcatheter Aortic Valve Replacement *LVEF*; Left Ventricular Ejection Fraction

The use of ProGlide and Manta was not randomized but performed consecutively, using first Proglide in 69 patients and then Manta in 75 patients, with only seven later ProGlide cases in between due to availability or operator preferences. This is reflected in some procedural variables, such as type of valve used and fluoroscopy time. Thus, seven Core Valves were implanted in the ProGlide group while none was used in the Manta group. Correspondingly, Evolut PRO was the most commonly used valve in the Manta group and Evolut R in the ProGlide group. The mean sheath size was similar between groups, whereas in the established Manta-use subgroup median sheath size was 16 Fr, as compared to 14 Fr in all other subgroups. The fluoroscopy time was significantly shorter in in the Manta established group compared to ProGlide established group, 18.9 ± 5.6 vs 24.1 ± 6.1 min, *p* < 0.001. For procedural variables, see Table 3.

**Table 3:** Procedural variables

	ProGlide learning (*n* = 25)	Manta learning (*n* = 25)	*p*	ProGlide established (*n* = 51)	Manta established (*n* = 50)	*p*
Valve type, n (%)						
Core Valve	5	0		2	0	
Evolut R	20	18		40	17	
Evolut PRO	0	7		9	33	
Sheath size (Fr)						
Mean ± SD	15.1 ± 1.8	15.4 ± 1.7	0.634	15.3 ± 1.7	15.5 ± 1.0	0.594
Median (range)	14 (14–18)	14 (14–20)		14 (14–18)	16 (14–18)	
Fluoroscopy time (minutes) mean ± SD	29.5 ± 5.4	27.9 ± 14	0.619	24.1 ± 6.1	18.9 ± 5.6	< 0.001
Contrast volume (milliliters) mean ± SD	171.8 ± 71.2	162.4 ± 52.4	0.597	147.0 ± 41.4	145.9 ± 52.1	0.914

*Fr* French, *SD* Standard Deviation

### Endpoints

There was no mortality attributed to access site bleeding in any of the groups.

The complication end point incidence (occlusion, bleeding, flow limiting dissection/stenosis and pseudo aneurysm) was significantly lower in ProGlide patients (*n* = 2; 2.7%), as compared to Manta patients (*n* = 8; 10.7%), *p* = 0.047. In the learning phase (patient 1–25 in each group) there was one complication in ProGlide group and 2 in the Manta group, *p* = 0.551, ns. In the established use ProGlide group there was one endpoint (2%) while in the established use Manta group there were 6 endpoints (12.0%), *p* = 0.047. Overall, the Manta complications consisted of bleeding (*n* = 6) and pseudo aneurysm (*n* = 4), Tables 4 and 5.

**Table 4:** Major vascular closure device / puncture site complications in ProGlide and in Manta

	ProGlide (*n* = 76)	Manta (*n* = 75)	*p*
All complications (occlusion, bleeding, dissection, stenosis or pseudo aneurysm), n (%)	2 (2.7)	8 (10.7)	0.047
Occlusion, n (%)	1 (1.3)	1 (1.3)	–
Bleeding, n (%)	2 (2.7)	6 (8.0)	–
Pseudo aneurysm, n (%)	0 (0)	4 (5.3)	–
Flow limiting stenosis or dissection, n (%)	1(1.3)	1 (1.3)	–

**Table 5:** Major vascular closure device / puncture site complications during learning and established procedure

	ProGlide learning (*n* = 25)	Manta learning (*n* = 25)	*p*	ProGlide established (*n* = 51)	Manta established (*n* = 50)	*p*
All complications (occlusion, bleeding, dissection, stenosis or pseudo aneurysm), n (%)	1 (4.0)	2 (8.0)	0.551	1 (2.0)	6 (12.0)	0.047
Occlusion, n (%)	1 (4.0)	0 (0)	–	0 (0)	1 (2.0)	–
Bleeding, n (%)	1 (4.0)	1 (4.0)	–	1 (2.0)	5 (10.0)	–
Pseudo aneurysm, n (%)	0 (0)	2 (8.0)	–	0 (0)	2 (4.0)	–
Flow limiting stenosis or dissection, n (%)	1 (4.0)	0 (0)	–	0 (0)	1 (2.0)	–

The endpoint of unplanned treatment (vascular surgery, stent/stentgraft/other vascular intervention) showed similar incidence in ProGlide patients (n = 2; 2.7%) and Manta patients (*n* = 7; 9.3%), *p* = 0.082. In the learning phase, the treatment endpoint occurred in one ProGlide patient (4.0%) and in one Manta patient (4.0%). However, in the established use phase, unplanned treatment occurred more frequently in Manta patients (n = 6; 12.0%) compared to ProGlide patients (*n* = 1; 2.0%), p = 0.047. Totally, there were two vascular interventions in two patients in the ProGlide group, while there were 8 vascular interventions in seven patients in the Manta group. For unplanned treatment details, see Tables 6 and 7. In the Manta group, there was one additional case of major access site bleeding treated by endovascular stentgraft. This bleeding was caused by accidental loss of guide wire access and could not be attributed to the VCD use and is therefore not included in the statistical analyses or in any endpoint.

**Table 6:** Vascular interventions in patients with VCD complications periprocedure and during hospital stay

	ProGlide (*n* = 76)	Manta (*n* = 75)	*p*
All interventions (vascular surgery, stent or stentgraft or other vascular interventions), n (%)	2 (2.7)	7 (9.3)	0.082
Periprocedural open vascular surgery, n (%)	1 (1.3)	1* (1.3)	–
Postprocedural open vascular surgery, n (%)	0 (0)	0 (0)	–
Periprocedural stent or stent-graft, n (%)	1 (1.3)	4* (5.3)	–
Postprocedural stent or stent-graft, n (%)	0 (0)	2 (2.7)	–
Other vascular intervention, n (%)	0 (0)	1 (1.3)	–

^*^One patient had both a stentgraft and open vascular surgery. VCD; vascular closure device

**Table 7:** Vascular interventions in patients with VCD complications during learning and in established procedure

	ProGlide learning (*n* = 25)	Manta learning (*n* = 25)	*p*	ProGlide established (*n* = 51)	Manta established (*n* = 50)	*p*
All interventions (vascular surgery, stent or stentgraft or other vascular interventions), n (%)	1 (4.0)	1 (4.0)	1.000	1 (2.0)	6 (12.0)	0.047
Periprocedural open vascular surgery, n (%)	1 (4.0)	0 (0)	–	0 (0)	1 (2.0)	–
Postprocedural open vascular surgery, n (%)	0 (0)	0 (0)	–	0 (0)	0 (0)	–
Periprocedural stent or stent-graft, n (%)	0 (0)	0 (0)	–	1 (2.0)	4 (8.0)	–
Postprocedural stent or stent-graft, n (%)	0 (0)	0 (0)	–	0 (0)	2 (4.0)	–
Other vascular intervention, n (%)	0 (0)	1 (4.0)	–	0 (0)	0 (0)	–

*VCD* vascular closure device

None of the ten patients with access site / VCD complications developed cardiac failure or permanent worsening of renal function. Eight of the ten patients had blood transfusions. In general, the present patients with complications had prolonged hospital stay. In all, including our hospital and secondary hospital or rehabilitation the median hospital stay was 9 (6–22) days.

## Discussion

The present study represents data from a non-randomized single centre registry on safety of two mechanically distinct types of percutaneous vascular closure devices (VCD) for arterial access site hemostasis in TF-TAVI. Our data suggests that there is fewer vascular access and closure device associated complications when using the presuture based ProGlide system compared to the recently introduced collagen based Manta system. Consequently, there was also a tendency for increased need for vascular surgery or endovascular intervention in the Manta group compared to the Proglide group. Furthermore, there was no decrease in frequency of complications in the Manta cohort, when transitioning from learning phase into established method phase. Both the frequency of serious complications and need for urgent or subacute vascular surgery or endovascular intervention was significantly higher in the established use Manta subgroup compared to the established use ProGlide subgroup. The composite complication endpoint in the Manta cohort was mainly driven by major bleeding and by pseudoaneurysm development. In none of the groups there was any mortality that could be attributed to access site bleeding.

Although the study groups were not randomized, they were not significantly different when comparing important demographics including age, STS/ACC TAVR risk score, body mass index (BMI), frequency of diabetes, hypertension and renal function. However, there were significantly more women in the ProGlide group vs the Manta group and there was more calcification in the pelvic arteries in the ProGlide patients. Further, the CFA diameter was smaller in the established ProGlide group compared to the established use Manta group. Interestingly, both female gender and a low CFA/sheath size ratio are known risk factors for access site complications (Dencker et al. 2016). However, these baseline differences should rather be in favour for the Manta cohort and disfavor for the ProGlide cohort.

During the inclusion time for the present study, there has been a continuous refinement of the TAVI procedure and the operators have gained more experience, and in parallel there has also been introduced new transcatheter heart valves from Medtronic, the Evolut XL and Evolut PRO. These valves are easier and faster to position compared to the initially used CoreValve, and thus contribute to the significantly shorter fluoroscopy time in the established use Manta group. The Manta itself also leads to very quick access site hemostasis, and thus contributes to shorter procedure times (Van Gils et al. 2016).

When Oslo University Hospital, Ullevål was established as a TAVI centre we performed one TAVI procedure every second week, which have gradually increased to 4–5 patients per week. Although initially the operator experience from TAVI was limited, all operators (AA-A, AO & PH) had long stand experience from vascular interventions and PCI. Also the increasing TAVI experience should, if anything, be in favour of the Manta group. Interestingly, the complication rate was similar between groups during learning, but remained high or even increased in the Manta established group. Our disappointing results with the collagen based Manta are in contrast with previous reports (Van Gils et al. 2016; Van Mieghem et al. 2017) and especially in contrast with two newly published registry studies in patient populations similar to ours by De Palma and co-workers (2018) and by Biancari and co-workers (2018), who reported high success rate and low bleeding and other complication rates when using Manta for access site closure in TAVI.

The Manta VCD is available in 14 and in 18 Fr size. In the study by Van Mieghem et al. (2017) 14 Fr Manta was used for sheath size up to 14 Fr and 18 Fr Manta up to 19 Fr sheath size. In the present study, 14 Fr Manta was accepted to be used in up to 16 Fr sheath size (personal communication with vendor representative and (Essential Medical, inc, 2018)). In the Manta cohort, and especially in the Manta established group, the most commonly used valve was the Evolut PRO, which requires 16 Fr sheath. In five of the eight reported access site related Manta complications, 14 Fr Manta VCD was used for closure of access site after using a 16 Fr introducer, with outer diameter of 5,3 mm. This miss-match in size may contribute to the higher rate of bleeding complications and pseudoaneurysms. Thus, until this is resolved one should use 18 Fr Manta for 16 Fr puncture sites.

Vascular complications and large bleeding are associated with renal failure, additional vascular surgery and endovascular interventions, to prolonged hospital stay and to increased mortality (Nara et al. 2017; Dencker et al. 2016), and access site complications constitute a major part of the total number of vascular complications during TF-TAVI. Thus, low complication rates are of uttermost importance for any new access site closure device introduced. Therefore, we believe that our work extends current knowledge on the use of the newly introduced Manta VCD compared to the well-established and well-documented Proglide VCD. Currently, the costs using Manta are substantially higher than for the ProGlide VCD. The further cost of surgery or intervention and prolonged hospital stay is an additional challenge.

In the present study, access site complications were preferably treated with stent or stentgraft rather than vascular surgery. Endovascular treatment has become widely accepted in this patient group due to the high age and fragility of these patients, and patency has been shown to be good (Segal et al. 2017).

### Limitations

The present data were obtained retrospectively from a single centre registry. The lack of randomization may influence selection of patients and outcomes of procedures. During the inclusion time, the TAVI procedure has been changed and refined, and there has also been a development in valves used, which influences the procedure and the introducer size. The grading of calcification in pelvic arteries was performed visually, and may thus have changed somewhat over the inclusion period. No non-access site vascular or other complications were analysed in the present patient group.

## Conclusions

In the present study of TF-TAVI access site complications the presuture based ProGlide VCD was associated with significantly lower rates of vascular complications and non-significant lower rates of urgent or subacute vascular surgery and endovascular interventions compared to the newly introduced collagen plug based Manta system. As our results are conflicting to other registry reports, a multicentre randomized trial of ProGlide vs Manta is suggested.

## Abbreviations

ACT: Activated Clotting Time
BMI: Body Mass Index
CFA: Common Femoral Artery
CT: Computed Tomography
Fr: French
SD: Standard Deviation
STS/ACC: Society of Thoracic Surgeons/American College of Cardiology
TAVI: Transcatheter Aortic Valve Implantation
TAVR: Transcatheter Aortic Valve Replacement
TF: Trans Femoral
VARC: Valve Academic Research Consortium
VCD: Vascular Closure Device
